# Pain in the Context of Family: A Study on Factors Contributing to Marital Satisfaction among Couples Suffering from Chronic Pain

**Published:** 2017-07

**Authors:** Fatemeh AKBARI, Mohsen DEHGHANI

**Affiliations:** 1. Family Research Institute, Shahid Beheshti University, Tehran, Iran; 2. Dept. of Psychology, Faculty of Psychology and Educational Sciences, Shahid Beheshti University, Tehran, Iran

**Keywords:** Chronic pain, Musculoskeletal, Family, Marital satisfaction, Couples

## Abstract

**Background:**

Chronic pain is associated with increased stress in families and has its own impact on the relationship between members of the family. Among couples, if one of them suffers from chronic pain, this may influence marital satisfaction in both of them and given the importance of marital satisfaction in the prediction of successful treatment outcome, it is important to investigate pain and personality-related factors contributing to inter-individuals variation in marital satisfaction.

**Methods:**

Participants in this study were recruited from Atieh Hospital and Rasa Pain Clinic, Tehran, Iran in the year 2013. Chronic pain patients and their spouses were asked to complete a battery of questionnaires including measures on pain cognition and marital satisfaction. Using correlation and regression analysis factors with the highest level of contribution to marital satisfaction were investigated.

**Results:**

Spouses’ level of rumination about patients’ pain has been found to be positively correlated with marital satisfaction in patients. In addition, patients’ depression has been found to be the best predictor of level of marital satisfaction in patients. In addition, patients’ level of depression, disability and perceived helplessness has been found to be the best predictors of marital satisfaction in their spouses.

**Conclusion:**

To improve marital satisfaction among chronic pain patients, their support-seeking needs should be satisfied through involvement of their spouses in the process of treatment. To improve marital satisfaction among patients’ spouses, we should help patients regain their ability, improve their mood, and make patients believe that they have access to the support of their caregivers.

## Introduction

Chronic pain condition exerts profound impact on both patients and their significant others, mainly spouses (1). Indeed, pain related disability adversely impacts family function, for it produces considerable role changes within the family system (2). Expectedly, such changes are likely to impose strain on family relationships, especially couple’s interactions, which is in congruence with the biopsychosocial model of pain (3, 4). According to this model, chronic pain condition not only decreases the wellbeing of chronic pain patients, but also adversely affects those who are in close relationship with them (5). For instance, patients and their spouses report lower rates of marital satisfaction once the pain becomes chronic (1). Several theories and studies place emphasis on the importance of marital relationships in chronic pain experience as such mutual relationships impact the well-being of both patients and their healthy spouses (1, 6), and increase the risk for chronic pain maintenance (7). For instance, spouses’ marital conflict has been shown to be associated with psychosocial impairment in both of them (2, 8). In addition, marital satisfaction has been shown to be associated with pain severity, physical disability, and depression in chronic pain patients (1, 9). More importantly, marital discord leads to greater display of pain behaviors in patients, which in turn results in more negative feelings in the spouses, and more punishing responses to pain (2, 8). On the other hand, higher levels of marital satisfaction protect the patients and their spouses against the negative outcomes related to chronic pain including mood disorders (6).

Given the existing literature and proposed important role of marital satisfaction in the enhancement of pain treatment outcomes, identifying factors that contribute to marital satisfaction in both patients and their spouses seems to be of great significance. Moreover, marital satisfaction decline in chronic pain patients and their spouses after the onset of pain condition (1, 2). Therefore, investigating factors that contribute to marital satisfaction is a promising avenue in pain literature. Different factors are associated with marital satisfaction among chronic pain patients and their spouses (1, 6). However, they have mostly focused on pain severity, physical disability, pain behaviors, and psychological distress as correlates of marital functioning (1, 6). Seemingly, the role of pain cognitions (i.e. pain catastrophizing: negative cognitive-affective response to anticipated or actual pain (10, 28) in predicting marital satisfaction is yet to be studied. Furthermore, the communal coping model of pain indicates that chronic pain patients communicate pain-related distress through catastrophic thoughts (11). Hence, it is important to investigate how such negative interpretations (i.e. pain catastrophizing) might affect their marital relationship. Hence, investigating how patients and spouses pain catastrophizing contribute to their marital satisfaction and determining whether such cognitions impose their effect beyond pain variables and depressive symptoms needs to be investigated. Individuals reporting higher levels of catastrophizing describe greater interpersonal problems as well (12). Therefore, chronic pain patients and their spouses’ catastrophizing might affect their levels of marital satisfaction. Indeed, patients who catastrophize might be more likely to report marital conflicts. In other words, patients who catastrophize are more likely to focus on the negative aspects of situations (i.e. marital relationship), and therefore, report lower levels of marital satisfaction (13).

The present study aimed to investigate factors that might contribute to both spouses’ marital functioning since pain and relationship difficulties are mutually related to one another.

## Methods

### Participants

Participants with musculoskeletal pain and their spouses (n=284) were recruited from Atieh Hospital and Rasa Pain Clinic, Tehran, Iran in 2013. The study was approved by University research Ethics Committee and Mental Health Center of Atieh Hospital.

Patients had to be older than 18 yr, be in constant pain for at least three months. Patients were excluded if they had brain injury or major cognitive dysfunction. All participants in this study provided informed consent and participated in the study voluntarily.

### Participant characteristics

Chronic pain patients were predominantly female (n=96, 67.6%) with an age average of 45.89 yr (SD=11.90). Their spouses were on average 47.51 yr old (SD=12.25). The couples had been married for a mean of 22.99 yr (SD=13.42). Chronic pain patients reported a mean pain duration of 46.33 (SD=65.69) months. The most common location for patients to experience pain was back and knee.

### Measures

Patients and their spouses completed a battery of questionnaires including pain intensity (VAS), fear of movement, pain catastrophizing, disability, depression, and Marital Adjustment Test (MAT). All measures showed good internal consistency –at or above 0.70 for the current study.

### Visual Analogue Scale (VAS)

The VAS is a 10 cm un-graded horizontal line with two anchors from 0 indicating “the minimum intensity of pain” to 100 indicating “the maximum intensity of pain”. Chronic pain patients were asked to indicate their current pain intensity on the scale, while their spouses were asked to indicate their interpretation of patients’ current pain. This scale has consistently demonstrated good validity and sensitive to change (14).

### Pain Catastrophizing Scale (PCS)

PCS is a 13-Item self-report scale that measures three dimensions of pain catastrophizing: Rumination, Magnification, and Helplessness. Respondents have to rank each statement on a 5-point Likert scale ranging from 0 (‘not at all’) to 4 (‘always’) with respect to past painful experiences and indicate the degree to which they experienced those thoughts and feelings (10). Chronic pain patients were administered the PCS (10), whereas their spouses were administered the significant other version of PCS (PCS-S) to assess the extent to which they catastrophize about patients’ pain (15). This measure has been translated into Persian and its psychometric properties are good (16, 17)]. Internal consistency (Cronbach’s alpha) for the PCS in the patients for the present sample was 0.86 for the 13-item total score, 0.71 for rumination, 0.66 for magnification, and 0.78 for helplessness. In addition, internal consistency (Cronbach’s alpha) for the PCS-S in the spouse sample was 0.80 for the 13-item total score, 0.71 for rumination, .64 for magnification, and 0.73 for helplessness.

### Tampa Scale of Kinesiophobia (TSK)

Fear of movement was assessed with Tampa Scale of Kinesiophobia, the patient and spouse versions (18, 19)]. It is a self-report scale including 17 items, each rated on a 4-point likert-type scale (1= extremely disagree, 4= extremely agree). Total score calculated after reversing 4 items (item numbers: 4, 8, 12, 16). Higher scores reflect greater fear of movement. This scale has good reliability and validity (19). This measure has been translated into Persian and its psychometric properties are good (16, 17). For the current study, inter-item reliability of this scale was good for both partners (pain spouses α=0.80; healthy spouses α=0.70).

### Roland and Morris Disability Questionnaire (RDQ)

The RDQ is a 24-item checklist designed to assess disability caused by pain. Patients were asked to mention whether each statement applied to them in the last 24 h. The RDQ score ranges from 0 (no disability) to 24 (maximum disability). In the current study, a modified version of RDQ was used for a heterogeneous group of chronic pain patients, the wording “my back pain” was changed to “my pain”, this modified version has shown good validity in pain samples (20). This measure has been translated into Persian and its psychometric properties are good (16, 17). Internal consistency reliability for RDQ in the present sample was 0.84.

### Depression Scale

Both spouses’ depressive symptoms were assessed with the depression subscale of DASS (21) which includes 14 items. Participants were asked to focus on the past week and report to what extent they have experienced each symptom on a Likert-type scale from 0 (did not apply to me at all) to 3 (Applied to me very much, or most of the time). This measure has been translated into Persian and its psychometric properties are good (16, 17). For the current sample, inter-item reliability of this subscale was excellent for both partners (pain spouses α=0.94; healthy spouses α=0.91).

### Marital satisfaction

Marital satisfaction was assessed by using Marital Adjustment Test (MAT). The 15-item MAT is commonly used as a measure of marital satisfaction. Greater scores indicate higher satisfaction, whereas lower scores reflect greater marital discord. Scores on the scale range from 2 to 158. Respondents are asked questions related to the degree of happiness with the marriage, agreement with the spouse on matters such as finances and the degree to which the respondent engages in outside activities with the spouse. The MAT has demonstrated good validity, inter-item reliability and test–retest reliability. For the current sample, internal consistency was good for both partners (pain spouses α=0.76; healthy spouses α=0.74).

## Results

### Correlations

Table 1 and 2 represent correlations between marital satisfaction, depressive symptoms, pain variables, and pain catastrophizing consecutively in patients and their spouses.

**Table 1:** Correlations between patients’ pain variables, pain catastrophizing subscales, and marital satisfaction

		**1**	**2**	**3**	**4**	**5**	**6**	**7**	**8**	**9**
1	VAS	-								
2	DASS	.06	-							
3	TSK	.18^*^	.44^**^	-						
4	Disability	.32^**^	.42^**^	.57^**^	-					
5	Rumination	.16	.36^**^	.35^**^	.31^**^	-				
6	Magnification	.20^**^	.57^**^	.41^**^	.21^*^	.35^**^	-			
7	Helplessness	.18^**^	.61^**^	.51^*^	.37^**^	.51^**^	.69^**^	-		
8	P.MAT	.09	−.55^**^	−30^**^	−.13	−.31^**^	−.26^**^	−.36	-	
9	S.MAT	.14	−.24^**^	−.06	.08	−.21^*^	−.11	−.26	.46^**^	-

N=284;

* *P*<0.01;

** *P*<0.0001

**Table 2:** Correlations between spouses’ pain variables, pain catastrophizing subscales, and marital satisfaction

		**1**	**2**	**3**	**4**	**5**	**6**	**7**	**8**	**9**
1	VAS	-								
2	DASS	.13	-							
3	TSK	.19^*^	.28^**^	-						
4	Disability	.16	.08	.22^**^	-					
5	Rumination	.09	.12	.17^*^	.01	-				
6	Magnification	.19^*^	.35^**^	.33^**^	.01	.21^*^	-			
7	Helplessness	.14	.36^**^	.30^**^	.05	.29^**^	.66^**^	-		
8	P.MAT	.02	−.27^**^	−.01	−.13	.22^**^	−.07	−.14	-	
9	S.MAT	−.004	−.52^**^	−.09	.08	.04	−.07	−.05	.46^**^	-

N=284;

* *P*<0.01;

** *P*<0.0001

Patients’ depressive symptoms, fear of movement, rumination, and magnification were correlated negatively and significantly with their marital satisfaction, showing that higher pain-related problems are associated with lower marital satisfaction in Patients. Patients’ rumination and depressive symptoms were negatively and significantly correlated with spouses’ marital satisfaction, showing that higher levels of depression and rumination about pain in patients are associated with lower levels of marital satisfaction in their Spouses. Pain intensity, disability, and helplessness were not significantly correlated with patients and spouses’ marital satisfaction.

Spouses’ depressive symptoms were negatively and significantly correlated with both spouses’ marital satisfaction, while spouses’ rumination was positively and significantly correlated with patients’ marital satisfaction. Higher levels of depression in caregivers is associated with lower levels of marital satisfaction for both patients and caregivers, while higher levels of rumination about patients’ pain is associated with higher levels of satisfaction in them. Spouses’ interpretation of pain intensity, magnification, and helplessness were not significantly correlated with both spouses’ marital satisfaction.

### Hierarchical regression: marital satisfaction

A hierarchical regression was used to test the hypothesis that pain beliefs (i.e. rumination, magnification, and helplessness) contribute unique variance to marital satisfaction over and above pain variables.

First, we examined the correlation between demographic variables (i.e. age, gender, yr of education, pain duration) and the dependent variable (marital satisfaction) to determine whether demographics should be entered as covariates. Gender was significantly correlated with healthy spouses’ marital satisfaction, but not patients’ marital satisfaction. Therefore, step 1 of the hierarchical regression for spouses’ marital satisfaction included education, step 2 consisted of pain variables (i.e. pain severity, fear of movement, and physical disability), step 3 consisted of depressive symptoms, and step 4 consisted of pain catastrophizing subscales (i.e. rumination, magnification, and helplessness).

As shown in Table 3 pain variables accounted for 11% of variance in patients’ marital satisfaction. Patients’ depressive symptoms contributed an additional 23% of the variance in patients’ marital satisfaction after controlling for pain variables.

**Table 3:** Hierarchical regression: patients’ marital satisfaction

**Patient variables**	**B**	**SE**	**Beta**	***t***
Step 1				
Pain severity	.17	.09	.14	1.73
Fear of movement	−1.37	.41	−.33	−3.32^**^
disability	.03	.53	.006	.06
		R^2^=.11^***^		
Step 2				
Depressive symptoms	−1.49	.22	−.54	−6.78^** *^
		R^2^=.34^***^		
Step 3				
disability	1.03	.48	.19	2.13^*^
Depressive symptoms	−1.55	.26	−.56	−5.86^***^
Rumination	−1.29	.70	−.15	−1.83
Magnification	1.39	.90	.15	1.54
Helplessness	−.29	.57	−.06	−.51
		R^2^=.37^***^		
Spouse variables				
Step 1				
Pain severity	.04	.10	.03	.40
Fear of movement	.05	.41	.01	1.36
disability	−.74	.45	−.14	−1.63
		R^2^=.02		
Step 2				
Depressive symptoms	−.88	.25	−.29	−3.47 ^***^
		R^2^=.10^**^		
Step 3				
Depressive symptoms	−.83	.26	−.28	−3.2^**^
Rumination	2.77	.81	.28	3.39^***^
Magnification	.65	1.10	.06	.59
Helplessness	−1.05	.62	−.18	−1.68
		R^2^=.18^***^		

* *P*<0.05;

** *P*<0.01;

*** *P*<0.0001

Patients’ disability and depressive symptoms accounted for 37% of variance in their marital satisfaction, whereas their pain catastrophizing did not contribute to their marital satisfaction. Spouse pain variables did not significantly contribute to patients’ marital satisfaction. Spouses’ depressive symptoms and rumination accounted for 18% of variance in patients’ marital satisfaction.

As shown in Table 4, patients’ disability, depressive symptoms, and helplessness accounted for 19% of variance in spouses’ marital satisfaction. In addition, spouses’ gender and depressive symptoms accounted for 35% of variance in their marital satisfaction, while spouse-related pain variables and their pain catastrophizing did not contribute to their marital satisfaction.

**Table 4:** Hierarchical regression: spouses’ marital satisfaction

**Patient variables**	**B**	**SE**	**Beta**	**t**
Step 1				
Gender	−9.26	4.5	−.17	−2.05^*^
		R^2^=.03^*^		
Step 2				
Pain severity	.11	.09	.10	1.16
Fear of movement	−.40	.43	−.10	−.94
Disability	.48	.51	.10	.96
		R^2^=.05		
Step 3				
Depressive symptoms	−.75	.23	−.29	−3.22^**^
		R^2^=.12^**^	-	
Step 4				
Disability	1.09	.51	.22	2.16 ^*^
Depressive symptoms	−.54	.28	−.21	−1.95 ^*^
Rumination	−1.15	.74	−.14	−1.54
Magnification	1.47	.95	.18	1.55
Helplessness	−1.36	.60	−.29	−2.27 ^*^
		R^2^=.19^***^		
Spouse variables				
Step 1				
Gender	−9.11	4.44	−.17	−2.05 ^*^
		R^2^=.02 ^*^		
Step 2				
Pain severity	−.009	.09	−.008	−.09
Fear of movement	−38	.38	−.08	−.98
Disability	.46	.41	.09	1.10
		R^2^=.04		
Step 3				
Gender	−8.71	3.85	−.16	−2.26^*^
Depressive symptoms	−1.52	.20	−.55	−7.42^***^
		R^2^=.32^***^		
Step 4				
Gender	−9.04	3.84	−.17	−.2.35^*^
Depressive symptoms	−1.67	.21	−.60	−7.77 ^***^
Rumination	.67	.67	.07	.99
Magnification	.20	.91	.02	.22
Helplessness	.72	.52	.13	1.38
		R^2^=.35^***^		

* *P*<0.05;

** *P*<0.01;

*** *P*<0.0001

## Discussion

This study aimed to test whether patients and their spouses’ pain catastrophizing contributed uniquely to their marital satisfaction after controlling other pain related variables correlated with marital satisfaction. Patient’ depressive symptoms accounted for an incremental variance in their marital satisfaction beyond that accounted for by pain variables, while their pain severity, rumination, magnification, and helplessness did not contribute to their marital satisfaction. Taken together, patients’ marital satisfaction is better explained by their depressive symptoms and disability. The role of depressive symptoms in predicting patients’ marital satisfaction as found in the current study is in congruence with the findings that depression has a significant impact on chronic pain patient’s relation satisfaction (22). That patients’ rumination, magnification, and helplessness did not significantly contribute to their marital satisfaction might be explained by this notion that patients’ negative beliefs are likely to indirectly affect marital satisfaction through their impact on other aspects of pain (i.e. depressive symptoms, and disability). The fear avoidance model of pain adequately explains such indirect relationships as pain-related catastrophizing leads to pain-related behaviors and consequent disability through the augmentation of fear of pain (23, 24). Physical disability caused by negative pain cognitions (i.e. pain catastrophizing) can be considered as a potential factor contributing to depressive symptoms and marital dissatisfaction in chronic pain patients. In addition, the results of this study revealed that spouses’ interpretation of patients’ pain did not contribute to patients’ marital satisfaction, whereas their rumination and depressive symptoms significantly contributed to patients’ marital satisfaction. Spouses’ beliefs considered as a contributing factor in patients’ psychological distress (15). Spouses with more depressive symptoms may fail to understand the emotional experience of the patients, and therefore, reveal less empathic behaviors to patients’ pain (25). Over time, patients’ feelings of not being understood by their spouses might contribute to their marital dissatisfaction.

Interestingly, spouses’ rumination was positively correlated with patients’ marital satisfaction. Such findings can be explained by the fact that content of the rumination items (i.e., “I keep thinking about how much it hurts my partner”) are representative of persistent thoughts about patients pain as well as spouses’ care and concern for patients’ health. Hence, rumination may result in a feeling of empathy and understanding of patients’ pain (pain affects spouses too), which may contribute to patients’ marital satisfaction as they are likely to receive more support and attention from their spouses.

Patients’ disability, depressive symptoms, and helplessness significantly explained spouses’ marital satisfaction, while their pain severity did not contribute to spouses’ marital satisfaction. Such findings lend support to the notion that pain-related disability is more likely to predict spouses’ marital satisfaction as it leads to functional impairment and might adversely affect family atmosphere through role changes. Indeed, pain adversely affects couples’ relationships as it gets chronic, but pain-related disability and its consequences are more likely to interfere with daily life. For example, helplessness beliefs are related to patients’ feelings of lack of control over pain that results in more avoidant behaviors, and disability (26). Therefore, disability and functional impairments, which are the consequences of helplessness thoughts, are likely to contribute to spouses’ marital dissatisfaction. Moreover, helplessness beliefs contribute to spouses’ marital satisfaction, as they will get hopeless for their not being able to help the patients (25, 28).

Spouses’ depressive symptoms explained a significant proportion of variance in their own marital satisfaction, while their interpretation of patients’ pain severity, fear of movement, rumination, magnification, and helplessness did not contribute to their marital satisfaction. Perhaps these variables have an indirect effect on spouses’ marital satisfaction by exacerbating depressive symptoms that seems to be a key factor contributing to marital satisfaction. Spouses who catastrophize are more likely to experience depressive symptoms since it is difficult for them to distract their attention from patients’ pain (1).

This study simultaneously examined the predicting role of both patients’ and spouses’ pain catastrophizing on their marital satisfaction while controlling for pain variables and depressive symptoms.

The current study may suffer from a number of limitations taken into account for the interpretation of findings. First, data for the current study gathered in one time point. Marital satisfaction is a dynamic concept that can change over the time dependent upon a number of variables (such as relationship with other members of family, financial status etc.). We were not able to repeat assessment in different time points and include all the other variables into account. In addition, we were not able to track changes in marital satisfaction over the time and to investigate its association with changes in pain cognition and mood of subjects. This also influenced our analyses and interpretation of findings of those analyses. In the future studies, longitudinal designs may help us to better investigate the causal relationship between the changes in mood and pain and changes in marital satisfaction.

## Conclusion

More focus on couples’ psychological states, especially their negative beliefs and depressive symptoms, may provide significant benefit in patients’ treatments through enhancing their marital satisfaction. Such outcomes seem to be a key step toward treatment enhancement as research shows that increase in marital satisfaction is related to better long-term treatment outcomes for both patients and their spouses. The findings of this study also place emphasis on the importance of considering spouses alongside patients both in pain research and in treatment to achieve better outcomes.

## Ethical considerations

Ethical issues (Including plagiarism, informed consent, misconduct, data fabrication and/or fal-satisfaction, double publication and/or submission, redundancy, etc.) have been completely observed by the authors.
